# ElectroMagnetoEncephalography Software: Overview and Integration with Other EEG/MEG Toolboxes

**DOI:** 10.1155/2011/861705

**Published:** 2011-03-15

**Authors:** Peter Peyk, Andrea De Cesarei, Markus Junghöfer

**Affiliations:** ^1^Department of Clinical Psychology and Psychotherapy, Saarland University, Campus, 66123 Saarbrücken, Germany; ^2^Department of Psychology, University of Bologna, 40127 Bologna, Italy; ^3^Institute for Biomagnetism and Biosignalanalysis, University of Münster, 48149 Münster, Germany

## Abstract

EMEGS (electromagnetic encephalography software) is a MATLAB toolbox designed to provide novice as well as expert users in the field of neuroscience with a variety of functions to perform analysis of EEG and MEG data. The software consists of a set of graphical interfaces devoted to preprocessing, analysis, and visualization of electromagnetic data. Moreover, it can be extended using a plug-in interface. Here, an overview of the capabilities of the toolbox is provided, together with a simple tutorial for both a standard ERP analysis and a time-frequency analysis. Latest features and future directions of the software development are presented in the final section.

## 1. Introduction

EMEGS (electromagnetic encephalography software) is an open-source software written in MATLAB and developed for the analysis of data collected with high density, whole head electroencephalography (EEG), and magnetoencephalography (MEG). It comprises batch functions to segment and filter continuous data, statistically exclude artifacts, correct eye movement artifacts, average across trials, interpolate for missing or noisy sensors, and average across participants. It offers a range of visualization modules for event-related potentials/event-related fields (ERP/ERF) curve plotting, wavelet spectrograms, data projection onto 3D models (sphere, realistic, or brain), and statistical coloring of *P*, *t* or *F* values. It allows to directly perform a variety of analyses and statistical tests on ERP/ERF data, such as inverse source estimations, current source density functions, MEG sensor position coregistration, fast Fourier transformation, principal component analysis, *t*-tests, repeated measures analysis of variance (ANOVA), permutation tests, regression, and correlation. It offers integration with *R* environment for statistical computing, allowing the calculation of advanced factorial designs directly on EEG and MEG data. 

This presentation of the software will be divided into three sections, which will provide an overview of the EMEGS project (Section 1), describe its analysis and visualization features (Section 2), and provide a perspective on future directions (Section 3). Additionally, two appendixes give simple tutorials on an ERP and on a wavelet analysis (Appendix A) and describe the use of plug-ins to extend EMEGS (Appendix B). 

### 1.1. Main Field of Software Application

Having been developed in a university environment, one main requirement of EMEGS is its applicability for both teaching and science—and thus for bachelor, diploma, master, and Ph.D. students, with not more than basic electrophysiological background knowledge and limited experience with EEG/MEG data analysis, as well as for experienced researchers demanding more than basic analysis functions. While some EEG/MEG analysis methods do not necessarily depend on a deep comprehension of their underlying functions and can be applied rather automatically, successful applications of various methods (e.g., EEG/MEG combination, realistic head modeling, integration of a priori knowledge in inverse modeling, Independent Component Analysis, etc.) strongly depend on the users experience. In fact, usage of more sophisticated analysis methods, in the absence of convergent knowledge and experience, does often result in less reliable study results compared to the application of more basic methods. This might be commonplace for all research areas but appears, at least from our experience, of particular importance for the field of EEG/MEG data analysis. Therefore, a “basic” interface and an “expert” interface have been developed in EMEGS, in order to fulfill the user-specific needs.

In its “basic mode,” the program allows novice users to thoroughly and autonomously analyze electrophysiological group studies, within the usually rather short amount of time given to prepare a thesis. It has thus been designed to be fast, easy to use, and easy to learn. It is completely based on graphical user interfaces, allows storage/loading of all relevant study parameter settings, and provides log files of all analysis steps and settings for final revision. The program tries to prevent typical novice user errors by giving warning messages and suggestions in case of rather unusual parameter settings or atypical combinations of methods (e.g., baseline correction of statistical values). It provides a pipeline for all major data analysis steps beginning with data preprocessing up to the final statistical analysis and visualization of results integrated within one program, avoiding the time consuming and, especially for beginners, often error-prone application of different analysis software components. All processing steps can be performed in batch mode, that is, a multitude of listed files can be processed identically and automatically. This does not only fasten but also secures the analysis against user errors (e.g., typos of parameter settings, which may have serious consequences especially in group studies). However, preventing a “black-box-” like usage, visual data inspection remains mandatory during the data artifact detection procedure and can be bypassed in “expert mode” only.

In the “expert mode,” the program provides a wider spectrum of functions and combinations thereof. For all analysis steps, EMEGS offers state of the art methods. However, the variety of methods is limited—for example, the L2-Minimum-Norm is provided as fundamental inverse method [1] but other inverse functions like LORETA, SWARM, Beamformer, or others are not. If a specific method is missing, such as estimations of functional and effective connectivity or boundary or finite element conductor models, EMEGS offers interfaces for data exchange with other commercial and noncommercial programs like Curry, Besa, BrainVision, SPM, FieldTrip and R (see below).

EMEGS has been developed and optimized for the analysis of group studies investigating distributed neural network activity with limited a priori knowledge of its spatial and temporal characteristics. EMEGS is not recommended if integration of very detailed a priori knowledge, such as number and location of underlying neural sources, is desired. EMEGS does not provide detailed localization of neural activities in individuals, such as localization of epileptic spikes. As it comes without any warranty (see below) EMEGS should not be used for clinical purposes.

### 1.2. Project History and People

The development of EMEGS was initiated in 1997 by Markus Junghöfer, who at that time was situated at Konstanz University (Germany), in order to analyze data collected with a 128-sensor Electrical Geodesics Incorporated (EGI) EEG device and a 4D-Neuroimaging/BTi 148 sensor MEG magnetometer system, but it has evolved since to support more data formats and analysis types. After Peter Peyk (Saarland University, Germany) joined the developer team in 2003, EMEGS was initially published under the terms of the GNU General Public License (GPL) in 2004 [2] and has since continued to appeal to a small but growing set of users. Substantial programming contributions have also come from Andrea De Cesarei (Bologna, Italy), Andreas Keil (Gainesville, USA), and Andreas Wollbrink (Münster, Germany), and the package implements subroutines from a number of other authors, namely Thomas Gruber (Osnabrück, Germany), Olaf Hauk (Cambridge, Great Britain), and Nathan Weisz (Konstanz, Germany). As all software developers were and are actively doing research in various fields of affective and cognitive neuroscience with varying methodological core areas, EMEGS has strongly been shaped in the direction of applicability in these fields.

### 1.3. Availability, License, and Support

**Figure 1:**
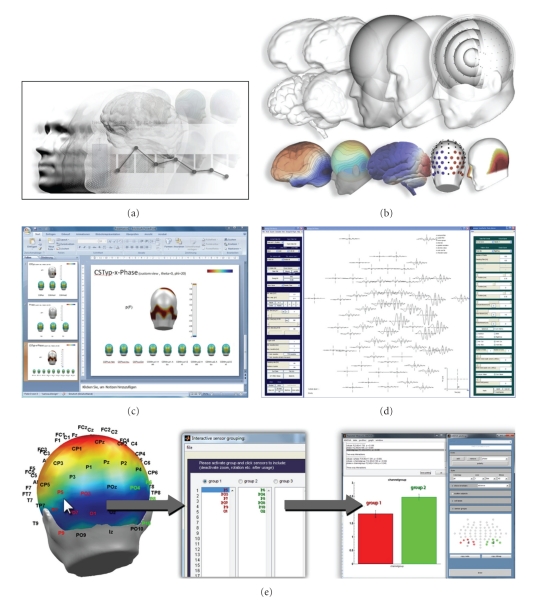
EMEGS snapshots of typical applications: (a) logo from the EMEGS web site at http://www.emegs.org/. (b) Various examples for the 3-dimensional visualization of results. (c) An automatically created Microsoft PowerPoint presentation as statistical report on a pointwise ANOVA. (d) The “Synthetic Data” graphical user interface was used to simulate a chirp signal. (e) Interactive sensor selection for statistical analysis with the built-in repeated measures ANOVA.

EMEGS is available for download free of charge at http://www.emegs.org/ (Figure 1(a)) and is managed as a CVS (Concurrent Version System) repository by a server located at Saarland University. Interested developers can apply informally for CVS access by email to the corresponding author. EMEGS is published under the terms of the GNU General Public License (GPL) v3. Documentation and help are provided in several manners. The program itself contains a documentation of the most frequent functions, while supplementary information is provided by online documentation. An email discussion list and an email archive exist at https://lists.sourceforge.net/lists/listinfo/emegs-user to allow users and developers to discuss problems and suggestions concerning the software, report bugs, and provide help. Furthermore, a revised chronological manual, written and continuously updated by novice users, will soon be available online. It will provide a standard operation procedure for the most common line of data analysis and give answers to frequently asked novice questions.

### 1.4. Supported Data Formats

EMEGS offers import/export or data conversion functions for averaged event-related potential or event-related field data sets from BESA, Vision Analyzer, Curry, EGI, Neuroscan, Biosemi, CTF, BTI, and European data format. All analysis tools on averaged data (see Section 2.3) are supported for EEG and MEG data equally. 

Full data preprocessing (i.e., filtering, epoching, artifact detection/extraction/correction, and averaging; see Sections 2.1 and 2.2) of continuous EEG data is supported for Electrical Geodesics Incorporated (EGI), Neuroscan continuous (CNT), European data format (EDF), and Biosemi data format (BDF). The import of data epoched in foreign software packages (epoch file formats by EGI, Neuroscan, etc.) for continued preprocessing in EMEGS (artifact detection and averaging) is not supported. 

Preprocessing of MEG data (continuous or epoched in foreign software packages) is not supported.

### 1.5. System Requirements

EMEGS is written in MATLAB and cannot run without the MATLAB environment (7.1 or higher). Moreover, for full functionality, it requires the MATLAB Signal Processing and Statistics Toolbox. EMEGS runs on almost all platforms that MATLAB can be installed on but has been tested most thoroughly on Windows and Linux. For statistical analyzes or group studies, with many participants, experimental conditions, channels, and time points, a large amount of RAM is required (>2 GB). Hardware accelerated graphics are helpful for 3D displays.

## 2. Features and Implementation

Once raw EEG or MEG data have been collected, several preprocessing steps have to be carried out. In particular, for each trial a data interval has to be selected in the time domain (segmentation around an event of interest) and in the frequency domain (high, low, or band-pass filtering). Additionally, data which have been contaminated by undesired events, such as muscular activity, electrical noise, or bad electrode contact, have to be detected. The original uncontaminated signal may be recovered using data correction techniques (e.g., eye movement correction) or sensor interpolation based on sensors containing uncontaminated data (as in the case of one single noisy sensor). Finally, experimental condition averages, across single and groups of participants, are calculated. With clean brain responses available, second-order analysis is usually sought after, such as source localization, wavelet and exploratory and inferential statistical analysis. For method testing and educational purposes, it can be useful to generate synthetic EEG/MEG data.

The following sections will give an overview of how these different tasks are implemented in EMEGS. Additionally, Appendix A provides a tutorial showing how a typical EMEGS analysis session is structured.

### 2.1. Preprocessing

Data preprocessing in EMEGS is optimized for statistical control of artifacts. It guarantees optimal signal-to-noise ratios with objective and identical parameter settings for all participants and experimental conditions within group studies. 

First, continuous interleaved data are converted to a demultiplexed format, to allow a fast and optimal filtering of channels by avoiding edge filter artifacts and allowing high-pass filtering with low cutoff frequencies. Biosemi data are re-referenced to Cz (Data in a Biosemi recording are written to file, referenced to the common mode sense (CMS) electrode and need to be re-referenced to remove the CM signal (c.f. http://www.biosemi.com/)), non-EEG/MEG channels are excluded, and the status (trigger) channel is analyzed for value changes that are written to a text file for faster access.

Second, continuous data are filtered as specified by the user. Visualization tools to investigate transfer functions of a variety of filters (Butterworth, Kaiser, firls, etc.) and identify reasonable filter settings are provided.

Third, trigger-based trials are extracted and stored in an epoch file, which contains trials in chronological sequence. An additional condition file in text format stores the trigger information of each epoch. Editing this file allows for reordering of experimental conditions. Special editing programs provide typical recoding types, such as balancing of trial numbers and odd-even split.

Fourth, if selected by the user, epochs are corrected for eye movement and eye blink artifacts, by either using a built-in implementation of the Gratton et al. regression approach [3] or by calling on the MATLAB-based toolbox BIOSIG [4], which contains a similar routine.

### 2.2. Artifact Detection and Averaging

After the preprocessing steps described above, parameters for the statistical editing of artifacts are calculated, saved to file, and applied. Typical parameters are the absolute maximum amplitude, the standard deviation, and the absolute maximum temporal gradient of potential/fields at individual trials, sensors and within or across predefined time intervals. Further parameters, such as amplitudes for specific frequency bands (e.g., alpha waves), may be added. These parameters serve to detect artifacts that are either global or specific to individual channels. Artificial sensors will be interpolated if the residual sensor distribution allows a reasonable approximation. The goodness of sensor interpolation is tested by interpolating a multitude (number of sensors) of sensor specific synthetic potentials or field topographies. If many to be interpolated sensors fall into one region or if many noisy sensors are positioned at the edge of the sensor coverage, a larger number of these test topographies are not interpolated with a sufficient decency and the corresponding trials get rejected from further analysis. For MEG or single-reference EEG data, statistical parameters are calculated and applied only once. For average reference EEG data however, this process is done by first using the recording reference and, in a second loop, using average reference [5]. The first pass avoids contamination of all channels by sensor-specific artifacts when transforming EEG data to average reference. The second pass, based on the average reference, detects global artifacts more clearly because the reference bias has been removed. The user interface to perform this statistical artifact detection is resented in more detail in the ERP tutorial section (Appendix A).

Following the artifact detection stage, data from each individual trial may fall in one of three scenarios. (1) If data from all sensors are clean, then all data will be averaged together to obtain the corresponding ERP/ERF. (2) If data from too many channels in one specific trial are too noisy and the above described interpolation check would indicate an insufficient goodness of interpolation, the trial will be discarded. (3) With a positive interpolation check, artifact-contaminated sensors within individual trials will be replaced by spherical spline interpolation, statistically weighted on the basis of all remaining sensors [6]. In this way, clean and approximated epochs are averaged by experimental condition in time or frequency domains and stored trial-by-trial on request for an optional second-order analysis (e.g., time-frequency analysis, wavelet, and single-trial inverse modeling).

### 2.3. Interpolation, Current Source Density, and Source Localization

To estimate brain signal values between sensor positions for mapping of surface models, EMEGS uses an inverse-forward source estimation [7], which can also be used to calculate the current source density (CSD) or Laplacian. Thus, the stiffness of the spherical spline functions, used for interpolation and CSD, is based on physiological parameters.

For source localization, EMEGS uses the L2-Minimum-Norm-Pseudoinverse (L2MNP), an inverse modeling technique, which estimates cortical generator structures without any a priori assumptions regarding the location and/or number of current sources [8, 9]. The classical minimum norm solution is a highly recommended inverse method, especially when no reliable a priori information about source generators is available [1]. As source model, a 4-shell (with radii of 2, 4, 6, and 8 cm) sphere model is used comprising 3 (azimuthal, polar, and radial) × 655 (EEG) or 2 (azimuthal, polar) × 655 (MEG) equidistant dipoles with a user adjustable Tikhonov regularization parameter *λ*. The outer two shells can also be used as separate full source models. The 4-shell source model is illustrated in Figure 1(b).

While visualization tools allow the representation of activations over realistic head or brain models, it should be emphasized that EMEGS does not provide inverse modeling routines based on realistic MRT- or CT-based head modeling. The 3D visualization tools, which are presented later on, are spherical projections from the underlying sphere-fit-based solutions on a realistic shaped head or brain surface model and are only used to illustrate the approximate localization of inverse solutions. In fact, although a sphere is a good first approximation of the human brain, there are quite strong deviations from the sphere model, especially at prefrontal cortex regions. Thus, the sphere-based localization of estimated neural activity deviates stronger from realistic head model-based estimations within these compared to other areas, providing a reasonable spherical fit [10]. It has to be noted though that the usage of realistic head models in inverse source estimations exhibits its own problems. As one example, radial sources do not result in measurable magnetic fields (MEG) outside of a sphere and can thus be ignored in spherical head models. Quasiradial sources in realistic models, however ask for cautious and user-dependent regularization, as they might otherwise provoke ghost effects as a mere consequence of inappropriate regularization. Again, there is no doubt that the usage of additional information, such as a MRI-, CT-, or DTI-based realistic volume conductor modeling, is advantageous with regard to the accuracy of inverse modeling. But usage of this additional information is still user dependent, that is, nonautomatic, and based on a deep understanding of the underlying algorithms—an understanding that, from our point of view, cannot be required from all software users.

### 2.4. Statistical and Exploratory Analysis of Evoked Brain Signals

A number of statistical and exploratory calculations on evoked or induced potentials/fields, as well as on their estimated underlying neural sources, can be run directly within EMEGS, without the need to export data to external statistical applications. Available calculations include various utility functions to calculate potential/field/source differences, averages, and EEG re-referencing, in addition to methods like principal component analysis, *t*-tests, correlation and regression analysis, repeated measures ANOVA, post hoc contrasts, permutation tests, global dissimilarity functions, spatial and temporal filtering, and so forth.

Most tests can either be done for defined sensor/source groups and selected time intervals or on all individual sensors/sources and all sample points. A graphical user interface for a fast and objective definition of sensor groups or source regions of interest and automatic identification of corresponding mirror groups/regions is provided, as is an interface for the interactive definition of analysis intervals. A useful application of sensor groups is the regional mean square calculation for a number of sensors, in order to obtain a regional power (e.g., the regional power of all occipital sensors). An application of interval means is, for instance, the creation of a statistical surface plot corresponding to a single region-of-interest ANOVA (e.g., the average potential from 80 to 120 ms after stimulus onset for the P1 component).

In addition to the repeated-measures statistical functions provided by MATLAB, EMEGS includes a built-in and graphical user interface-based repeated-measures ANOVA that can handle up to six within factors—including the two within-factors sensor/source groups and time intervals of interest—and up to three between factors. This ANOVA can be run as a single region-of-interest analysis with bar graphs and post hoc testing (illustrated in Figure 1(e)) or as a point-by-point analysis, resulting in 4d statistical parametric maps, that are stored in standard SCADS files and can be displayed like any other brain signal. All statistical methods allow various online data transformations during data import, such as averaging across time intervals or sensor groups, rectification, temporal and spatial filtering, or further custom made transformations. This allows a fast exploration on the impact of different parameter settings (e.g., spatial and temporal filtering, strength of regularization) or alternative data transformation methods (e.g., baseline correction versus high-pass filtering) on the final statistical results.

To control for the accumulation of type I errors, EMEGS provides post hoc filtering routines to scan significant time ranges for a sufficient sequence length (number of time points) and spatial spread (number of adjacent significant sensors) and remove results that do not match these criteria. The nonparametric permutation (rerandomization or exact) test is also provided.

### 2.5. Data Display and Visualization

EMEGS allows the visualizing of potentials, fields or estimated sources as well as statistics thereof (*F* value or *t* value distributions) as curves with planar projection of the sensor arrays, single sensor zoom, global power (GP), and global root-mean-square (RMS), global mean or corresponding time curves for sensor-groups or regions of interest definition. Global or regional power/RMS/mean values on a trial-by-trial basis can be visualized by time × trial color surface plots.

Data may also be visualized as 3D projections onto several models, including a simple sphere model, a realistic head shape, a realistic brain shape, and spatially smoothed versions of the former two (Figure 1(b)). For illustrative purposes, a brain shape comprised of independently selectable brain lobes has been implemented. Coloring can be selected from a large set of linear, exponential, logarithmic, or customized color maps and configured as smooth transitions or as contour plot type. Sensor/source positions or groups thereof can be displayed and colored at leisure. Visualization parameters such as line or surface coloring and line options can be stored and loaded for repetitive usage.

The temporal development of any 3-dimensional projection can be illustrated on a column- and rowwise subplot figure, using either default equidistant and consecutive time intervals or customized intervals with variable length and onsets. These four-dimensional illustrations can also be presented as movies and can be stored in various movie file formats.

Overview functions to compare corresponding averages of trial-by-trial data curves (2D) or data topographies (3D) for all participants and experimental conditions are provided (e.g., for identification of spurious effects in participants and/or experimental conditions). Additionally, several other data types may be visualized, including horizontal scrollable raw data display, spectrogram wavelet display, and magnetic resonance imaging (MRI) data.

Statistical results may be exported as continuous waveforms (4d-SPMs) and projected on a 3D head model. Statistical graphs may be created in parametric mapping mode, highlighting significant regions and time ranges, and hiding areas where nonsignificant results were observed.

In addition, EMEGS offers a number of functions to automate the creation of surface plots, for instance for the purpose of visual inspection across an entire sample of experimental conditions and/or subjects or for statistical reports. It can automatically create a Microsoft PowerPoint presentation after a point-by-point ANOVA has been run—with cell averages and *P* value maps brought together on one slide per main effect and interaction (illustrated in Figure 1(c)).

### 2.6. Generation of Synthetic Data

For educational purposes, as well as for method development testing, EMEGS also provides a generation of synthetic EEG/MEG data based on default or customized sensor distributions. Location and direction of single or multiple synthetic neural sources with various predefined or customized waveforms can be defined. Various combinations of temporally uncorrelated (white), temporally correlated (Gaussian), spatially uncorrelated (sensor noise), and spatially correlated noise (brain noise), as well as simulated ocular artifacts, are provided. Simulated data can be stored in various averaged or single-trial formats. Signal and noise amplitudes can be systematically varied across simulated trials or simulated averaged participant data.  Figure 1(d) visualizes the corresponding “Synthetic Data” graphical user interface (left and right frame) used here to simulate sweep or chirp signals (central frame). Such signals can for instance be used to illustrate or test the application of time-frequency methods, such as wavelet analysis.

### 2.7. Extending EMEGS Capabilities: Exemplified Time Frequency Analysis Using FieldTrip

The modulation of brain activity, which is related to an event of interest, can be investigated using event-related potentials or fields. However, this technique reflects modulations of neural correlates with rather strong phase coupling to an event of interest [11]. Therefore, a growing interest has arisen towards techniques that allow the examination of signals with rather weak phase coupling. Analysis of time-frequency characteristic EEG/MEG data allows one to observe the extent to which specific rhythms (e.g., alpha or gamma) are modulated by experimental events, regardless of their phase orientation [12]. This analysis separates phase and power information [13], and EEG/MEG spectral changes can be classified as phase locked (*evoked*) or nonphase locked (*induced*). 

To allow time-frequency analysis of electrophysiological data demanding more than the basic functions provided by EMEGS, an EMEGS plug-in (see Appendix B) has been recently added, which integrates EMEGS preprocessing with functions provided by the FieldTrip EEG processing toolbox [14]. At a basic level, EMEGS and FieldTrip serve the same purpose: to review EEG/MEG data, analyze them, and visualize and export the results. At a more specific level however, each application offers its users a selected choice of analysis algorithms and graphical user interfaces (GUIs). Here, a plug-in is described, which calls on FieldTrip routines to perform a time-frequency analysis on trial-by-trial EMEGS data, running through EEGLAB [15] for data conversion. 

All analysis steps (data conversion, parameter selection, and analysis) are handled by the plug-in GUI, which provides settings for a reasonable set of parameters allowing to perform a Morlet wavelet analysis. GUIs for the setting of further wavelet parameters could be added easily. The plug-in interface allows to adjust wavelet parameters (width and length) and calculate time-frequency changes in power and in the phase-locking factor. The Laplacian or Current Source Density (CSD) may be applied as additional deblurring methods [7, 16, 17] for a better identification of regional EEG/MEG spectral changes. In addition, a variable frequency resolution can be used, and results can be written in a FieldTrip- or EMEGS-readable format. Finally, simple operations (grand average, average, and difference) can be performed on the result files. Results can be visually displayed using the provided visualization plug-in, or using the standard EMEGS visualization functions. The use of the EMEGS time-frequency plug-in is illustrated in Appendix A.

## 3. Future Directions

Here, an overview of the EMEGS processing capabilities was given in terms of data preprocessing, statistical analysis, and data visualization. As an example of the EMEGS plug-in architecture, an integration with the FieldTrip software to perform time-frequency analysis was presented. 

Analyzing electromagnetic brain signals represents a special challenge, as EEG/MEG modulations reflect brain processes with spatial, temporal, and functional properties that are largely unknown. New analysis approaches are suggested as research advances, and they allow the uncovering of specific characteristics of neurophysiological signals. It is common to all developers of EEG/MEG data analysis software to implement these new methods in their applications or to provide convenient interfaces to other software that provides specific functions.

EMEGS is continuously being developed, and new functions are added on a regular basis. For instance, work on an EEG/MEG beamformer approach for source modeling is in progress (see applications in [10]). Statistical capabilities are being extended by integrating EMEGS with the R software for statistical computing [18]. (For the analysis of between-group designs with unequal group sizes, EMEGS calls the R software for statistical computing (available at http://www.r-project.org/) via the R-(D)COM Interface *Statconn* (available at http://www.statconn.com/) on Windows and the R-package *Rserve* and Java R client *JRClient* (available at http://rosuda.org/Rserve/) on UNIX-type operating systems.) Additionally, a stand-alone version of EMEGS (http://www.emegs.org/wiki/emegs qt version) is under construction (preliminary releases of the Qt version are already available for download on the EMEGS website (http://www.emegs.org/)), programmed in C++ and built with the open-source cross-platform GUI library Qt (http://qt.nokia.com/). This version is meant to provide the conveniences of modern graphical user interfaces, which cannot be realized within MATLAB, and allows for faster openGL-based graphics. Furthermore, analysis modules for peripheral psychophysiological measures, such as electrocardiography (ECG) and electrodermal activity (EDA), are under construction.

As an open-source project, EMEGS welcomes participation of new developers. To this end, the plug-in architecture provides an easy way to extend EMEGS analysis capabilities, while maintaining an easy to use graphical interface. Here, a plug-in devoted to run a Morlet wavelet analysis was described. It is important to note that, using the mechanisms and low-level functions described here, users can quite easily create new plug-ins, for instance to access the analysis functions provided by EEGLAB or FieldTrip directly from the EMEGS interface.

## 4. Conclusion

In sum, we recommend the usage of EMEGS as a fast and secure pipeline for EEG/MEG data analysis. EMEGS provides all relevant data analysis modules from preprocessing to statistics and final visualization of results within one integrative package. Users with only basic methodological knowledge may particularly benefit as EMEGS allows them to investigate typical neuroscientific research questions in a rather easy, fast, secure, and autonomous way. As such, EMEGS especially recommends itself for researchers with quite limited temporal resources and restricted assistance from advanced users to learn and apply software packages offering a greater variety of analysis tools. However, EEG/MEG experts also benefit from the highly automated and standardized analysis pipeline. For specific nonprovided functions, EMEGS offers interfaces for data exchange with other commercial and noncommercial EEG/MEG analysis software packages.

## Figures and Tables

**Figure 2:**
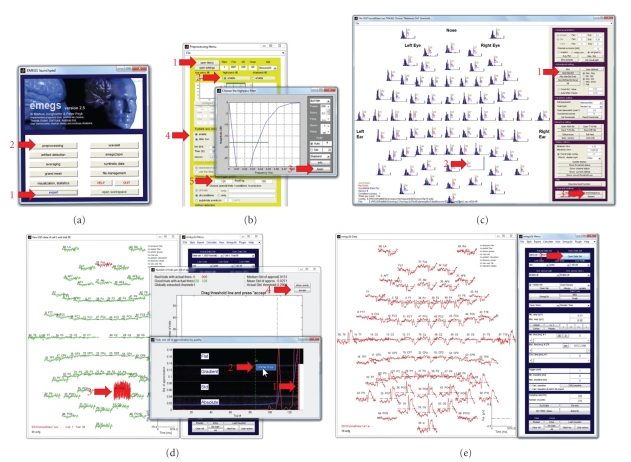
ERP tutorial illustration. Red arrows indicate processing steps of the tutorial. (a) The EMEGS launch pad, which can be used to start all major EMEGS programs. (b) The EMEGS preprocessing program, with an additional dialog to configure a high-pass filter. (c) The EMEGS artifact detection program, which is based on statistical parameter distributions. (d) Detailed display of single trial data during artifact threshold editing. (e) The EMEGS program for 2D ERP/ERF data display.

**Figure 3:**
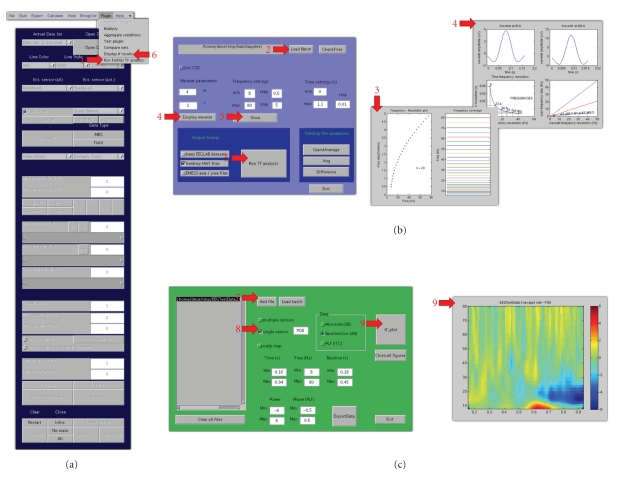
Time-frequency tutorial illustration. Red arrows indicate processing steps of the tutorial. (a) The EMEGS program for 2D ERP/ERF data display, illustrating the plug-in menu. (b) The plug-in analysis interface, including diagnostic plots. (c) The plug-in visualization interface, including analysis results from sensor PO8.

**Algorithm 1:**
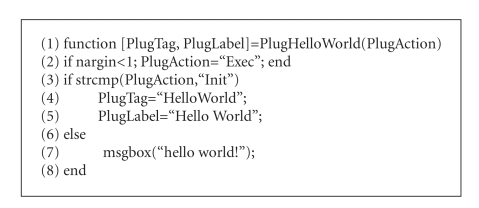


## Appendices

### A. EMEGS Analysis Tutorial

In this section, two brief analysis tutorials will be given to illustrate the EMEGS processing facilities described above. A 70-sensor EEG data file acquired at 256 Hz with a simple passive viewing paradigm was used in this tutorial. It includes 44 trials in each of three experimental conditions (3 different neutral faces), presented in random order. The processed data is included in the EMEGS download (at …/emegs2.5/emegs2dTestData/eeg/BinData/Biosemi/EEGTutorialData.7z) as is a more detailed version of this tutorial, which interested users are encouraged to consult for following along (http://www.emegs.org/tutorial.html). To run this tutorial, EMEGS ≥2.5 must be extracted in the Matlab path. To run the wavelet analysis, FieldTrip and EEGLAB must also be downloaded and extracted in the Matlab path.

#### A.1. ERP Analysis Tutorial

##### A.1.1. Preprocessing

Open the EMEGS start dialog (called “EMEGS launch pad”, Figure 2(a)) by typing “emegs” in the MATLAB command window. Activate the “expert” mode (the expert mode is needed to run the eye movement correction using the BIOSIG toolbox files) on the bottom of the launch pad (Figure 2(a)-1), then start the “Preprocessing” tool (Figure 2(a)-2). A yellow interface will open (Figure 2(b)), push the “open data file” button at the top (Figure 2(b)-1) and select the demo datafile in the file dialog. Enable a 0.1 Hz high-pass filter (Figure 2(b)-2) by clicking on the “enable” button under “high pass filt” and then selecting “Apply” (Figure 2(b)-3). To perform an automatized eye movement correction, activate the eye blink/eye movement correction button (Figure 2(b)-4), then activate the checkbox on the bottom of the preprocessing interface reading “eyecorr with biosig” (not shown). To select the size of the data epochs to be extracted, set the number of points to be extracted before each trigger to 125 (Figure 2(b)-5, “PreTrig”) and the number of points to be extracted after each trigger to 150 (Figure 2(b)-5, “PostTrig”). Finally, push the “Run” button on the bottom of the preprocessing window (not shown) to start the analysis. EMEGS will start the preprocessing and will write a log file in the folder where the data file resides (called “PrePro.log”).

##### A.1.2. Artifact Detection

Continue with the artifact detection by pushing the “Edit processed files” button on the bottom of the preprocessing window (not shown). To start the artifact detection, push the “Abs MaxStd” button (Figure 2(c)-1), which has EMEGS performance statistical control of artifacts based on the maximum absolute amplitude (Abs) and the standard deviation excluding outliers on the maximum side of the trial distribution (MaxStd). The program will then display histograms of the Abs and MaxStd parameters for each channel (Figure 2(c)). Narrow unimodal distributions in nearly all cases indicate good data quality, whereas multimodal and broad distributions nearly always indicate a substantial amount of artifacts in the data. EMEGS prompts you to set the trial threshold separating good from bad trials manually (Figure 2(d)) by dragging the dotted red line (Figure 2(d)-1) from left to right to the proper position. Each column of the gray background graph codes the parameters of one trial, and columns (trials) are sorted by data quality with the best trials on the far left and the worst trials on the far right. The dotted red line you need to adjust marks the threshold between good and bad trials. For the present data file, data quality is very good, so a far right position indicating mostly good trials and very few bad trials is appropriate. To check the data of a specific trial, right click and select “show trial” from the context menu (Figure 2(d)-2). The data display program comes up and shows the current trial, highlighting good channels in green and bad channels in red (Figure 2(d)-3). When you have set the threshold properly, push the “accept” button in the window named “Number of trials per std of approx.:” (Figure 2(d)-4). A graphical display of the chronological sequence of good and bad trials is displayed next (not shown), and global and trial- and channel-specific thresholds are written to disk.

##### A.1.3. Event-Related Averaging, Sensor Interpolation, and Simple Data Display

Push the “Close all and EmegsAvg” button on the artifact detection main window (Figure 2(c)-3) to directly start the averaging window for the edited data. The data format is set automatically, and you just need to push the “Run average” button to start the averaging and approximation of missing sensors.

Now, you should be able to find the result files in the data folder. Namely, the files “EEGTutorialData.f.ses.app1,” “EEGTutorialData.f.ses.app2,” and “EEGTutorialData.f.ses.app3” containing the clean data epochs for the conditions 1, 2, and 3, and the files “EEGTutorialData.f.at1.ar,” “EEGTutorialData.f.at2.ar,” and “EEGTutorialData.f.at2.ar” containing the ERPs. Single-trial and averaged data was converted to average reference.

Push the “Close All and Emegs2d” button on the bottom right corner of the averaging window, and the data display and analysis window will open (Figure 2(e)). Push the “Open data file” button (Figure 2(e)-1) and select one of the ERP-files listed above to display its data.

##### A.1.4. Wavelet Analysis

Run the time-frequency analysis plug-in from Emegs2d -> Plug-in -> Run Fieldtrip TF analysis, or run *Plugtfwltgui* from the Matlab prompt (Figure 3(a)-1). In the plug-in interface (Figure 3(b)-2), push the “load *.app*” button and select any of the three newly created .app* files. Now enter the wavelet analysis parameters: under the “frequency settings” label, set min = 8, step = 0.5, max = 80, and step = 5, to focus on data from 8 Hz to 80 Hz. Steps between successive frequencies now vary linearly from 0.5 Hz for the lowest frequencies to 5 Hz for the highest frequencies. Which and how many frequencies will be analyzed can be displayed by pushing the “show” button (Figure 3(b)-3). Since the pretrigger and the posttrigger interval is rather short in the present data, wavelet parameters will be set to m = 4 and c = 2, to obtain a short wavelet which can be repeated several times in the data segment, even at the lowest frequencies. In general, longer baseline and data segments allow for more accurate time-frequency analyses. To display the resulting wavelet, push the “display wavelet” button (Figure 3(b)-4). In the “time settings” section, specify min = 0, max = 1.1, and step = 0.01 to analyze the entire 1100 ms epoch in steps of 10 ms each. Push “Run TF analysis” to start the analysis (Figure 3(b)-5). Data will be automatically converted in the EEGLAB format, then imported in Fieldtrip, and analyzed using the chosen parameters. As a result, a .mat file containing the time-frequency results, in terms of power and phase-locking factor (PLF), will be created in the same folder as the original data.

To display results, choose Emegs2d -> Plug-in -> Display TF results (Figure 3(a)-6) or run *Plugtfgui* from the MATLAB command window. Load the newly created .mat files by selecting “Add file” (Figure 3(c)-7), then activate “single sensor” from the visualization options and “PO8” as sensor of interest (Figure 3(c)-8). In the time section, enter min = 0.16, max = 0.94, with baseline correction from 0.18 to 0.45 (as described in the ERP tutorial, data epochs include 500 ms of baseline, thus stimulus onset is at 0.5 s). Change the power scale to −6 to +6, and select tf_plot to display a Fieldtrip plot of the chosen sensor (Figure 3(c)-9). In a similar fashion, the whole-head topographies and multiple sensor plots can be easily obtained for both power and PLF data.

### B. EMEGS Plug-ins

Since EMEGS version 2.0, the facility to create plug-ins has been added. A plug-in is a MATLAB script that is placed in the “… emegsX.X/emegs2dUtil/Plug-ins” directory. This directory is automatically analyzed at EMEGS startup, to create menu entries for each plug-in. Plug-ins can also be directly executed as MATLAB commands, while EMEGS is not running. Algorithm 1 is the basic structure of a plug-in.

At startup, EMEGS scans the plug-in directory and executes each plug-in with the optional argument PlugAction = “Init”. The plug-in interprets this as an EMEGS request and outputs a tag and a file menu descriptor, which will be used by EMEGS to create the corresponding menu entries (lines 3–5). On the other hand, when the corresponding menu entry is selected, or when the plug-in is executed from the MATLAB prompt with no arguments, the code indicated at line 7 is executed (in the example, a message box with the text “hello world!” is displayed). EMEGS plug-ins offer an easy way to integrate ones own analysis functions into the EMEGS GUI. Therefore, they were chosen here to integrate EMEGS, EEGLAB, and FieldTrip analysis functions.
